# Supplementary material for: D-serine reconstitutes synaptic and intrinsic inhibitory control of pyramidal neurons in a neurodevelopmental mouse model for schizophrenia
Source: Nat Commun. 2023 Dec 12;14:8255. doi: 10.1038/s41467-023-43930-8 (PMC10716516; doi:10.1038/s41467-023-43930-8)
Supplement: Supplementary file 1 — Supplementary Information [file 41467_2023_43930_MOESM1_ESM.pdf]

Supplementary Information to:

D-serine reconstitutes synaptic and intrinsic inhibitory control of pyramidal neurons in a neurodevelopmental mouse model of schizophrenia

Xiao-Qin Zhang [1], Le Xu [1], Xin-Yi Zhu [1], Zi-Hang Tang [1], Yi-Bei Dong [1], Zhi-Peng Yu [1], Qing Shang [2], Zheng-Chun Wang [1], Hao-Wei Shen [1, 2]*

1. Department of Pharmacology, School of Medicine, Ningbo University, 818 Fenghua Rd, Ningbo, Zhejiang 315211, China

2. Department of Neurology, The First Affiliated Hospital of Ningbo University, 59 Liuting Street, Haishu District, Ningbo, Zhejiang 315211, China

*Correspondence

Dr. H.W. Shen,

Department of Pharmacology

School of medicine, Ningbo University, 818 Fenghua Rd, Ningbo, Zhejiang, 315211, China

Tel: +86-574-87609585; Fax +86-574-87608638

Email: shenhaowei@nbu.edu.cn

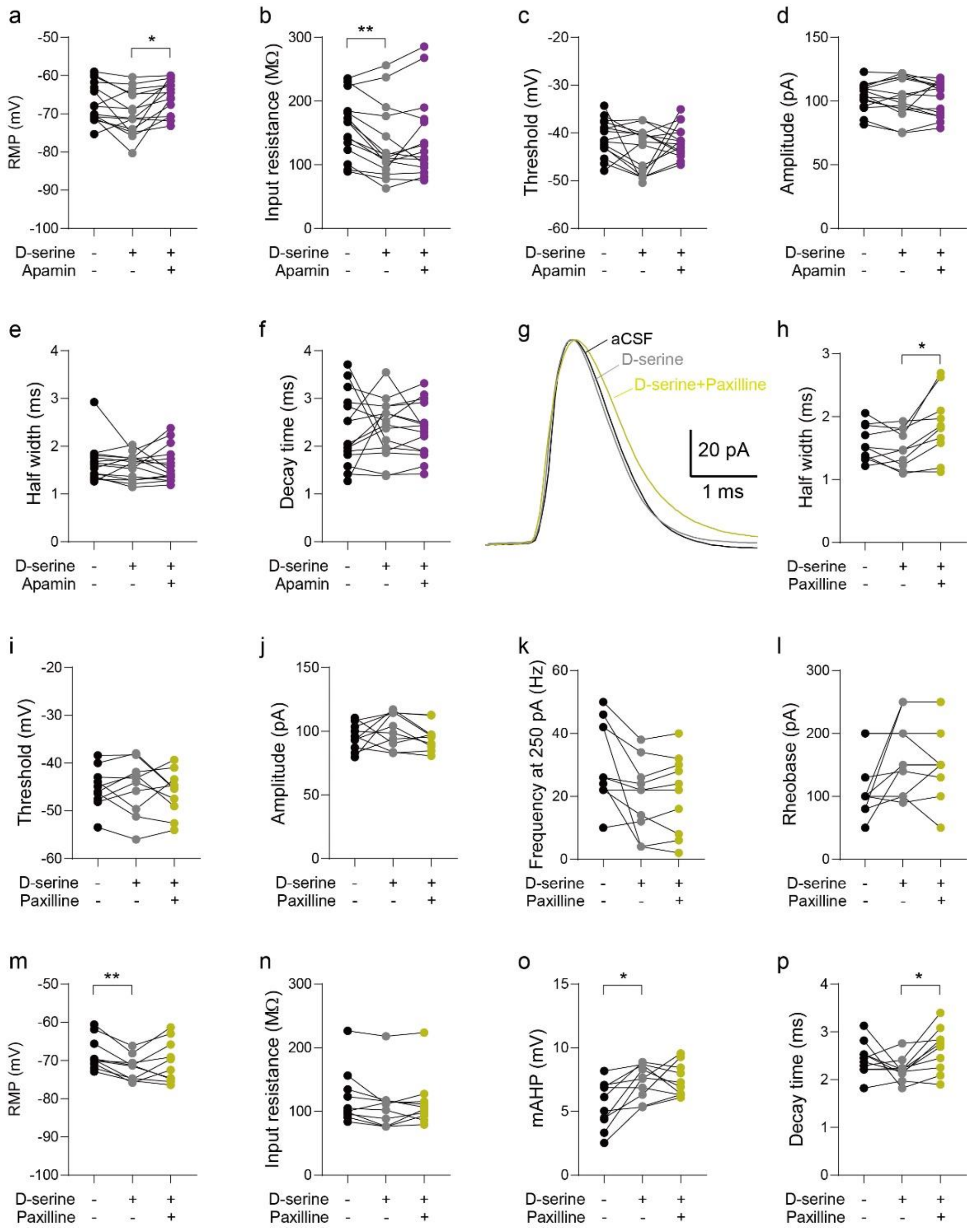

Supplementary Fig. 1 The effect of D-serine on the electrophysiological properties of pyramidal neurons in the ACC of control group, related to Fig. 1.

a-f The effect of D-serine and SK channel blocker (apamin) on RMP (**a**), input resistance (**b**), threshold (**c**), amplitude (**d**), half width (**e**) and decay time (**f**) from pyramidal neurons. *p = 0.0123, **p = 0.0026, Bonferroni's post hoc test following one-way ANOVA (n = 15 neurons from 5 mice). **g** Representative of AP waveforms in pyramidal neurons before (black), after D-serine (grey) or D-serine + Paxilline (yellow). **h-p** The effect of D-serine and BK channel blocker (paxilline) on half width (**h**), threshold (**i**), amplitude (**j**), firing frequency in response to 250 pA current (**k**), rheobase (**l**), RMP (**m**), input resistance (**n**), mAHP (**o**) and decay time (**p**) from pyramidal neurons. *p = 0.0197 (**h**), *p = 0.0124 (**o**), *p = 0.0155 (**p**), **p = 0.0017, Bonferroni's post hoc test following one-way ANOVA (n = 10 neurons from 4 mice). Source data are provided as a Source Data file. mAHP, medium afterhyperpolarization; RMP, resting membrane potential.

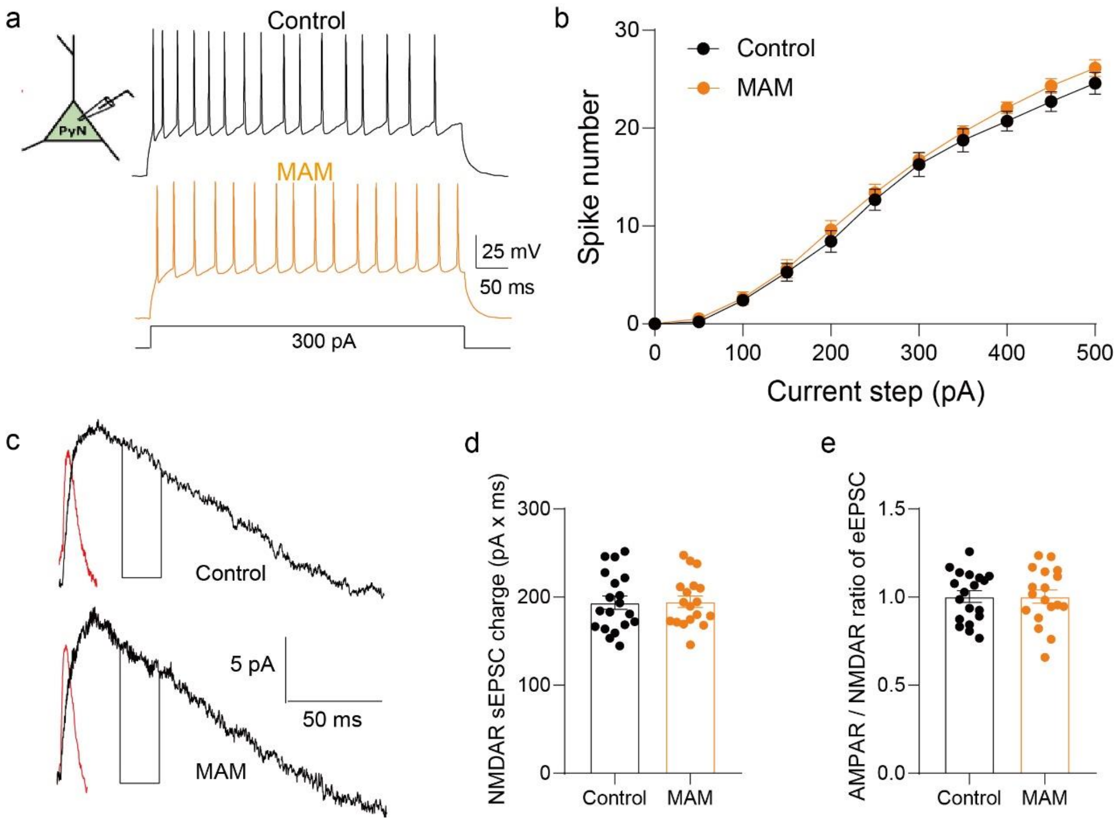

Supplementary Fig. 2 No significant difference in NMDAR function and excitability of pyramidal neurons in the ACC between control and MAM group, related to Fig. 3.

a Representative traces of action potential and from pyramidal neurons in control and MAM group, respectively. **b** The spike number of pyramidal neurons in MAM group (Control: n = 29 neurons from 10 mice, MAM: n = 31 neurons from 10 mice; two-way ANOVA: MAM $F_{(1, 58)} = 0.683$, $p = 0.412$). **c** The diagram showed typical average events of NMDAR-sEPSC in pyramidal neuron in the ACC. NMDAR-sEPSCs were estimated from the charge of the dual sEPSCs after AMPAR contribution decayed to zero. Dotted lines represent the time window where the NMDAR-sEPSCs charge were calculated. **d** NMDAR-sEPSC charge of the pyramidal neurons (Control: n = 19 neurons from 10 mice, MAM: n = 18 neurons from 10 mice; two-sided unpaired *t* test: $t_{(35)} = 0.104$, $p = 0.918$). **e** AMPAR/NMDAR ratio of sEPSC in the pyramidal neurons (Control: n = 19 neurons from 10 mice, MAM: n = 18 neurons from 10 mice; two-sided unpaired *t* test: $t_{(35)} = 0.008$, $p = 0.994$). Data are shown as mean ± SEM. Source data are provided as a Source Data file.

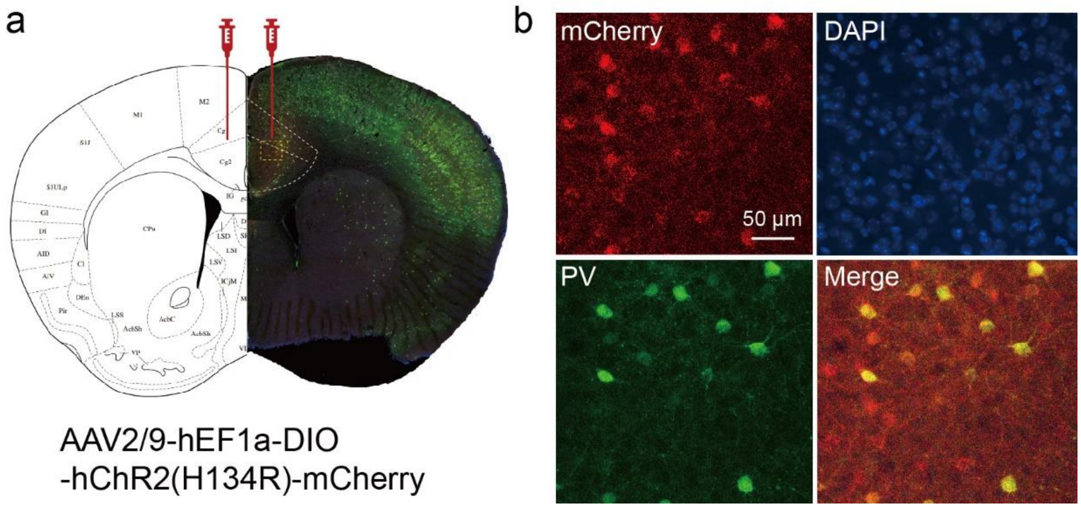

Supplementary Fig. 3 Opotogenetic activation of PV neurons in control or MAM groups, related to Fig. 4.

a Image of AAV expressing AAV2/9-hEF1a-DIO-hChR2(H134R)-mCherry microinjected into the ACC of PV-cre mice. **b** ChR2-mCherry was specifically expressed in PV neurons. This experiment was repeated 3 times with similar results.

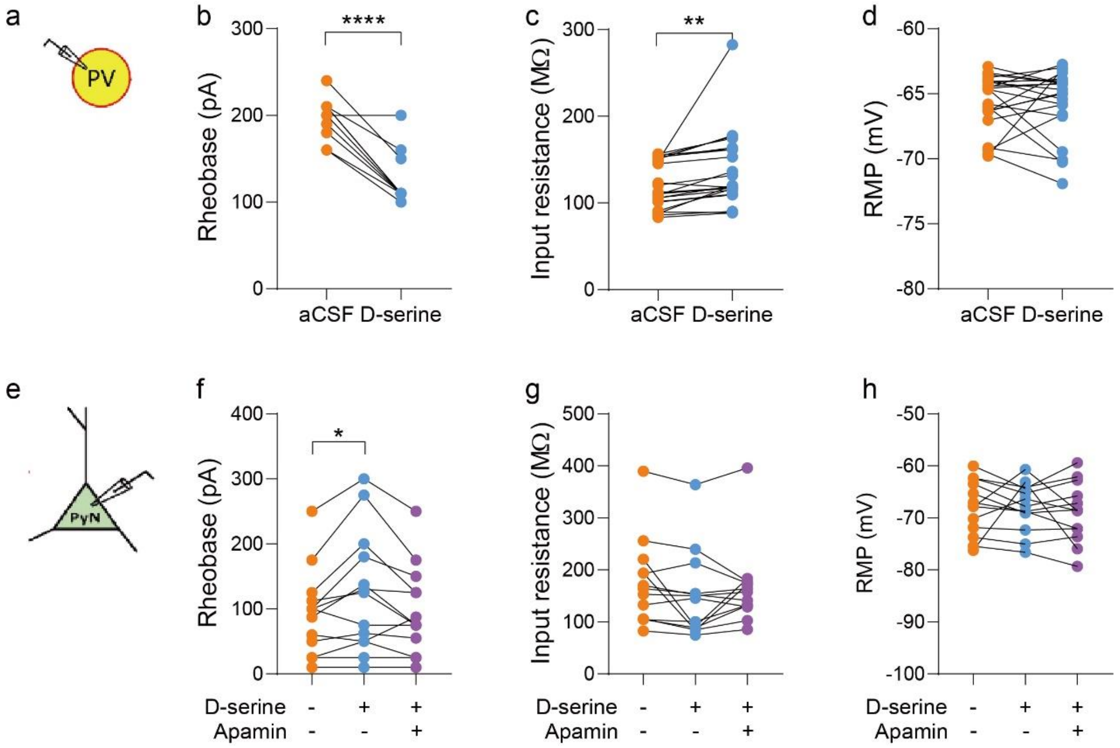

Supplementary Fig. 4 The effect of D-serine on the electrophysiological properties of PV or pyramidal neurons in the ACC of MAM group, related to Fig. 5.

a Diagram of whole-cell recording of PV neuron. **b-d** D-serine significantly decreased rheobase (two-sided paired t-test: $t_{(9)} = 7.10$, ****$p < 0.0001$; n = 10 neurons from 4 MAM group) and increased input resistance (two-sided paired t-test: $t_{(22)} = 3.037$, **$p = 0.0061$; n = 23 neurons from 8 MAM group) in PV neurons. **e** Diagram of whole-cell recording of pyramidal neuron. **f-h** The effect of D-serine on rheobase, input resistance and RMP of pyramidal neurons. *$p = 0.0383$, Bonferroni's *post hoc* test following one-way ANOVA (n = 13 neurons from 5 mice). Source data are provided as a Source Data file. RMP, resting membrane potential.

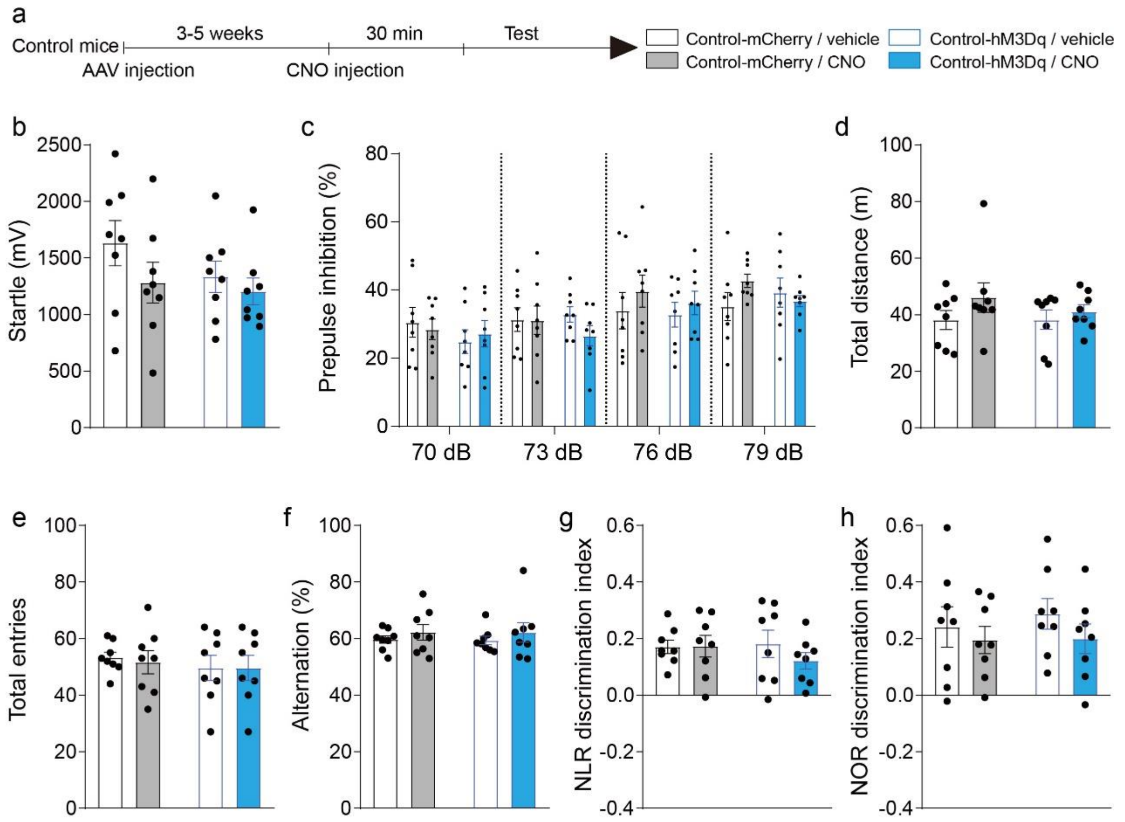

Supplementary Fig. 5 No effect of chemogenetic activation of PV neurons in the ACC of control group, related to Fig. 7.

a Timeline of AAV injection and CNO or vehicle administration in PV-cre control mice. **b & c** Selective activation of PV neurons showed no significant effect on acoustic startle and PPI inhibition in MAM group (Two-way ANOVA). **d-h** No significant effect of activating PV neurons on locomotor activity in OFTs, spatial working memory in Y-maze tests and recognition memory in NLR and NOR tests (two-way ANOVA). n = 8 in each group. Data are shown as mean ± SEM. Source data are provided as a Source Data file. CNO, clozapine N-oxide; NLR, novel location recognition; NOR, novel object recognition.

Supplementary Table 1: the number of neurons and mice used in each group

Figure	Experiment	Number of cells	Number of mice	Statistics
Fig. 1c	Spike recording of pyramidal neuron	25	8 (4M, 4F)	two-way ANOVA
Fig. 1e	Spike recording of PV neuron	11	4 (2M, 2F)	two-way ANOVA
Fig. 1g-i	Spike recording of pyramidal neuron	15	5 (3M, 2F)	one-way ANOVA
Fig. 1k-m	Spike recording of PV neuron	11	4 (2M, 2F)	Paired t test
Fig. 1n	Spike recording of PV neuron	14	6 (3M, 3F)	two-way ANOVA
Fig. 1p	eAP recording of pyramidal neuron	23	6 (3M, 3F)	two-way ANOVA
Fig. 1q	eAP recording of pyramidal neuron	23	6 (3M, 3F)	one-way ANOVA
Fig. 2b-c	PPI	/	Control / vehicle: 12 (6M, 6F); Control / D-serine: 8 (4M, 4F); MAM / vehicle: 13 (7M, 6F); MAM / D-serine: 10 (5M, 5F)	two-way ANOVA
Fig. 2d	OFT	/	Control / vehicle: 13 (7M, 6F); Control / D-serine: 8 (4M, 4F); MAM / vehicle: 13 (7M, 6F); MAM / D-serine: 10 (5M, 5F)	two-way ANOVA
Fig. 2e-f	Y maze	/	Control / vehicle: 13 (7M, 6F); Control / D-serine: 8 (4M, 4F); MAM / vehicle: 16 (8M, 8F); MAM / D-serine: 10 (5M, 5F)	two-way ANOVA
Fig. 2g-h	NLR, NOR	/	Control / vehicle: 13 (7M, 6F); Control / D-serine: 8 (4M, 4F); MAM / vehicle: 13 (7M, 6F); MAM / D-serine: 10 (5M, 5F)	two-way ANOVA
Fig. 3b-f	Spike recording in PV neuron	Control: 21; MAM: 23	Control: 10 (5M, 5F); MAM: 10 (5M, 5F)	two-way ANOVA & Unpaired t test
Fig. 3i-j	NMADR-EPSC recording in PV neuron	Control: 20; MAM: 20	Control: 10 (5M, 5F); MAM: 10 (5M, 5F)	Unpaired t test
Fig. 4c-e	oIPSC recording in pyramidal neuron	Control: 20; MAM: 20	Control: 7 (4M, 3F); MAM: 7 (3M, 4F)	two-way ANOVA & Unpaired t test
Fig. 4g-h	sIPSC recording in pyramidal neuron	Control: 15; MAM: 13	Control: 6 (3M, 3F); MAM: 6 (3M, 3F)	Unpaired t test
Fig. 4j	eAP recording in pyramidal neuron	Control: 21; MAM: 19	Control: 6 (3M, 3F); MAM: 6 (3M, 3F)	Chi-square test
Fig. 5b-c	Spike recording in PV neuron	12	5 (3M, 2F)	two-way ANOVA & one-way ANOVA
Fig. 5d	Spike recording in PV neuron	Control: 11; MAM: 10	Control: 4 (2M, 2F); MAM: 4 (2M, 2F)	two-way ANOVA
Fig. 5e-f	NMADR-EPSC recording in PV neuron	Control: 13 or 10 (with D-serine); MAM: 13 or 16 (with D-serine)	Control: 6 (3M, 3F) or 5 (with D-serine, 3M, 2F); MAM: 6 (3M, 3F) or 6 (with D-serine, 3M, 3F)	two-way ANOVA
Fig. 5h-j	Spike recording in pyramidal neuron	13	4 (2M, 2F)	two-way ANOVA & one-way ANOVA
Fig. 5k	Spike recording in pyramidal neuron	Control: 13; MAM: 13	Control: 4 (2M, 2F); MAM: 5 (2M, 3F)	two-way ANOVA
Fig. 5l	eAP recording in pyramidal neuron	19	6 (3M, 3F)	one-way ANOVA
Fig. 6c &d	Spike recording in PV neuron	Control / vehicle: 16 MAM / vehicle: 18 MAM / D-serine: 16	Control / vehicle: 6 (3M, 3F) MAM / vehicle: 6 (3M, 3F) MAM / D-serine: 6 (3M, 3F)	two-way ANOVA & one-way ANOVA
Fig. 6f	eAP recording in pyramidal neuron	Control: 20; MAM: 19	MAM / vehicle: 6 (3M, 3F); MAM / D-serine: 6 (3M, 3F)	Chi-square test
Fig. 7d-f	Spike recording in PV neuron	13	4 (2M, 2F)	two-way ANOVA & paired t test
Fig. 7h-i	PPI	/	MAM-mCherry / vehicle: 10 (5M, 5F); MAM-mCherry / CNO: 10 (5M, 5F); MAM-hM3Dq / vehicle: 10 (5M, 5F); MAM-hM3Dq / CNO: 10 (4M, 6F)	two-way ANOVA
Fig. 7j	OFT	/	MAM-mCherry / vehicle: 8 (4M, 4F); MAM-mCherry / CNO: 8 (5M, 3F); MAM-hM3Dq / vehicle: 10 (5M, 5F); MAM-	two-way ANOVA

			hM3Dq / CNO: 10 (4M, 6F)	
Fig. 7k-l	Y maze	/	MAM-mCherry / vehicle: 8 (4M, 4F); MAM-mCherry / CNO: 8 (5M, 3F); MAM-hM3Dq / vehicle: 10 (5M, 5F); MAM-hM3Dq / CNO: 10 (4M, 6F)	two-way ANOVA
Fig. 7m-n	NLR, NOR	/	MAM-mCherry / vehicle: 8 (4M, 4F); MAM-mCherry / CNO: 8 (5M, 3F); MAM-hM3Dq / vehicle: 10 (5M, 5F); MAM-hM3Dq / CNO: 10 (4M, 6F)	two-way ANOVA
Fig. 8b-f	Spike recording in pyramidal neuron	aCSF group: 20; other each group: 14	aCSF group: 6 (3M, 3F); other each group: 5 (2M, 3F)	two-way ANOVA & one-way ANOVA
Fig. 8h-n	Behavioral tests	/	Control + vehicle: 8 (4M, 4F), Control + riluzole: 8 (4M, 4F), MAM + vehicle: 8 (4M, 4F), MAM + riluzole: 7 (4M, 3F)	two-way ANOVA
Fig. S1a-f	Spike recording in pyramidal neuron	15	5 (3M, 2F)	one-way ANOVA
Fig. S1g-p	Spike recording in pyramidal neuron	10	4 (2M, 2F)	one-way ANOVA
Fig. S2b	Spike recording in pyramidal neuron	Control: 29; MAM: 31	Control: 10 (5M, 5F); MAM: 10 (5M, 5F)	two-way ANOVA
Fig. S2d & e	NMADR-EPSC recording in pyramidal neuron	Control: 19; MAM: 18	Control: 10 (5M, 5F); MAM: 10 (5M, 5F)	Unpaired t test
Fig. S4b	Spike recording in PV neuron	10	4 (2M, 2F)	Paired t test
Fig. S4c & d	Spike recording in PV neuron	23	8 (4M, 4F)	Paired t test
Fig. S4f-h	Spike recording in pyramidal neuron	13	5 (3M, 2F)	one-way ANOVA
Fig. S5b-h	Behavioral tests	/	Control-mCherry / vehicle: 8 (4M, 4F); Control -mCherry / CNO: 8 (4M, 4F); Control -hM3Dq / vehicle: 8 (4M, 4F); Control -hM3Dq / CNO: 8 (4M, 4F)	two-way ANOVA

Supplementary Table 2: Sex-based analysis

Figure	Experiment	Number of cells	Number of mice	Statistics
Fig. 2b-c	PPI	/	Control: 12 (6M, 6F); MAM: 13 (7M, 6F).	two-way ANOVA:<br>**Startle:** MAM × Sex $F_{(1, 21)}$ = 0.495, p = 0.489; MAM $F_{(1, 21)}$ = 2.422, p = 0.135; Sex $F_{(1, 27)}$ = 0.175, p = 0.680.<br>**70 dB:** MAM × Sex $F_{(1, 21)}$ = 0.159, p = 0.694; MAM $F_{(1, 21)}$ = 41.08, p < 0.0001; Sex $F_{(1, 21)}$ = 0.353, p = 0.559.<br>**73 dB:** MAM × Sex $F_{(1, 21)}$ = 0.770, p = 0.390; MAM $F_{(1, 21)}$ = 24.81, p < 0.0001; Sex $F_{(1, 21)}$ = 2.312, p = 0.143.<br>**76 dB:** MAM × Sex $F_{(1, 21)}$ = 0.419, p = 0.525; MAM $F_{(1, 21)}$ = 5.913, p = 0.024; Sex $F_{(1, 21)}$ = 1.953, p = 0.177.<br>**79 dB:** MAM × Sex $F_{(1, 21)}$ = 0.143, p = 0.709; MAM $F_{(1, 21)}$ = 4.246, p = 0.052; Sex $F_{(1, 21)}$ = 0.773, p = 0.389.
Fig. 2d	OFT	/	Control: 13 (7M, 6F); MAM: 13 (7M, 6F).	two-way ANOVA<br>MAM × Sex $F_{(1, 22)}$ = 0.261, p = 0.615; MAM $F_{(1, 22)}$ = 1.819, p = 0.191; Sex $F_{(1, 22)}$ = 0.094, p = 0.762.
Fig. 2e-f	Y maze	/	Control: 13 (7M, 6F); MAM: 16 (8M, 8F).	two-way ANOVA<br>**Entries:** MAM × Sex $F_{(1, 25)}$ = 3.837, p = 0.061; MAM $F_{(1, 25)}$ = 0.123, p = 0.729; Sex $F_{(1, 25)}$ = 0.139, p = 0.712.<br>**Alternation:** MAM × Sex $F_{(1, 25)}$ = 0.022, p = 0.884; MAM $F_{(1, 25)}$ = 23.54, p < 0.0001; Sex $F_{(1, 25)}$ = 0.615, p = 0.440.
Fig. 2g-h	NLR, NOR	/	Control: 13 (7M, 6F); MAM: 13 (7M, 6F).	two-way ANOVA<br>**NLR:** MAM × Sex $F_{(1, 22)}$ = 0.010, p = 0.922; MAM $F_{(1, 22)}$ = 15.59, p < 0.001; Sex $F_{(1, 22)}$ = 0.003, p = 0.958.<br>**NOR:** MAM × Sex $F_{(1, 22)}$ = 0.028, p = 0.868; MAM $F_{(1, 22)}$ = 47.74, p < 0.0001; Sex $F_{(1, 22)}$ = 0.252, p = 0.621.
Fig. 3b-f	Spike (200 pA) recording in PV neuron	Control: 21; MAM: 23	Control: 10 (5M, 5F); MAM: 10 (5M, 5F)	two-way ANOVA<br>**Spike:** MAM × Sex $F_{(1, 40)}$ = 0.399, p = 0.531; MAM $F_{(1, 40)}$ = 5.194, p = 0.028; Sex $F_{(1, 40)}$ = 0.801, p = 0.376.<br>**Rheobase:** MAM × Sex $F_{(1, 40)}$ = 0.122, p = 0.729; MAM $F_{(1, 40)}$

				= 9.680, *p* = 0.003; Sex $F_{(1, 40)}$ = 0.284, *p* = 0.597.<br>**RMP:** MAM × Sex $F_{(1, 40)}$ = 0.012, *p* = 0.912; MAM $F_{(1, 40)}$ = 11.29, *p* = 0.002; Sex $F_{(1, 40)}$ = 0.231, *p* = 0.633.<br>**Resistance:** MAM × Sex $F_{(1, 40)}$ = 0.375, *p* = 0.544; MAM $F_{(1, 40)}$ = 10.87, *p* = 0.002; Sex $F_{(1, 40)}$ = 2.936, *p* = 0.094.
Fig. 3i-j	NMADR-EPSC recording in PV neuron	Control: 20; MAM: 20	Control: 10 (5M, 5F); MAM: 10 (5M, 5F)	two-way ANOVA<br>**Charge:** MAM × Sex $F_{(1, 36)}$ = 0.016, *p* = 0.901; MAM $F_{(1, 36)}$ = 16.62, *p* < 0.001; Sex $F_{(1, 36)}$ = 1.283, *p* = 0.265.<br>**Ratio:** MAM × Sex $F_{(1, 36)}$ = 0.052, *p* = 0.822; MAM $F_{(1, 36)}$ = 11.16, *p* = 0.002; Sex $F_{(1, 36)}$ = 0.014, *p* = 0.905
Fig. S2b	Spike recording (300 pA) in pyramidal neuron	Control: 29; MAM: 31	Control: 10 (5M, 5F); MAM: 10 (5M, 5F)	two-way ANOVA<br>MAM × Sex $F_{(1, 56)}$ = 0.878, *p* = 0.353; MAM $F_{(1, 56)}$ = 0.113, *p* = 0.738; Sex $F_{(1, 56)}$ = 0.151, *p* = 0.699.
Fig. S2d & e	NMADR-EPSC recording in pyramidal neuron	Control: 19; MAM: 18	Control: 10 (5M, 5F); MAM: 10 (5M, 5F)	two-way ANOVA<br>**Charge:** MAM × Sex $F_{(1, 33)}$ = 0.089, *p* = 0.767; MAM $F_{(1, 33)}$ = 0.007, *p* = 0.936; Sex $F_{(1, 33)}$ = 0.228, *p* = 0.636.<br>**Ratio:** MAM × Sex $F_{(1, 33)}$ = 0.490, *p* = 0.489; MAM $F_{(1, 33)}$ = 2.142e-007, *p* = 0.999; Sex $F_{(1, 33)}$ = 0.182, *p* = 0.673.
