# Supplementary material for: D-serine reconstitutes synaptic and intrinsic inhibitory control of pyramidal neurons in a neurodevelopmental mouse model for schizophrenia
Source: Nat Commun. 2023 Dec 12;14:8255. doi: 10.1038/s41467-023-43930-8 (PMC10716516; doi:10.1038/s41467-023-43930-8)
Supplement: Supplementary file 3 — Reporting Summary [file 41467_2023_43930_MOESM3_ESM.pdf]

nature portfolio

Corresponding author(s): Hao-Wei Shen

Last updated by author(s): Nov 6, 2023

Reporting Summary

Nature Portfolio wishes to improve the reproducibility of the work that we publish. This form provides structure for consistency and transparency in reporting. For further information on Nature Portfolio policies, see our Editorial Policies and the Editorial Policy Checklist.

Statistics

For all statistical analyses, confirm that the following items are present in the figure legend, table legend, main text, or Methods section.

n/a	Confirmed	
☐	☒	The exact sample size (*n*) for each experimental group/condition, given as a discrete number and unit of measurement
☐	☒	A statement on whether measurements were taken from distinct samples or whether the same sample was measured repeatedly
☐	☒	The statistical test(s) used AND whether they are one- or two-sided<br>*Only common tests should be described solely by name; describe more complex techniques in the Methods section.*
☐	☒	A description of all covariates tested
☐	☒	A description of any assumptions or corrections, such as tests of normality and adjustment for multiple comparisons
☐	☒	A full description of the statistical parameters including central tendency (e.g. means) or other basic estimates (e.g. regression coefficient) AND variation (e.g. standard deviation) or associated estimates of uncertainty (e.g. confidence intervals)
☐	☒	For null hypothesis testing, the test statistic (e.g. *F*, *t*, *r*) with confidence intervals, effect sizes, degrees of freedom and *P* value noted<br>*Give P values as exact values whenever suitable.*
☒	☐	For Bayesian analysis, information on the choice of priors and Markov chain Monte Carlo settings
☒	☐	For hierarchical and complex designs, identification of the appropriate level for tests and full reporting of outcomes
☒	☐	Estimates of effect sizes (e.g. Cohen's *d*, Pearson's *r*), indicating how they were calculated

Our web collection on statistics for biologists contains articles on many of the points above.

Software and code

Policy information about availability of computer code

Data collection	Electrophysiology data were collected using pCLAMP version 10.7 software (Molecular Devices). Behavioral data were collected using TopScan v2.0 (Xiaomi, Inc.).
Data analysis	Electrophysiology data were analysed using Clampfit version 10.7 (Molecular Devices). Pre-pulse inhibition (PPI) tests behavioural analyses were performed using acoustic startle response system (AniLab Scientific Instruments, Ningbo, China). Open field tests and novel location recognition (NLR) and novel object recognition (NOR) behavioural analyses were performed using ANY-maze software (Stoelting). Statistical analysis was performed using Graphpad Prism version 8 (GraphPad Software, San Diego, CA).

For manuscripts utilizing custom algorithms or software that are central to the research but not yet described in published literature, software must be made available to editors and reviewers. We strongly encourage code deposition in a community repository (e.g. GitHub). See the Nature Portfolio guidelines for submitting code & software for further information.

Data

Policy information about availability of data

All manuscripts must include a data availability statement. This statement should provide the following information, where applicable:

- Accession codes, unique identifiers, or web links for publicly available datasets
- A description of any restrictions on data availability
- For clinical datasets or third party data, please ensure that the statement adheres to our policy

All relevant data supporting the key findings of this study are available within the article and its Supplementary Information files. Source data are provided with this paper.

Research involving human participants, their data, or biological material

Policy information about studies with human participants or human data. See also policy information about sex, gender (identity/presentation), and sexual orientation and race, ethnicity and racism.

Reporting on sex and gender	N/A
Reporting on race, ethnicity, or other socially relevant groupings	N/A
Population characteristics	N/A
Recruitment	N/A
Ethics oversight	N/A

Note that full information on the approval of the study protocol must also be provided in the manuscript.

Field-specific reporting

Please select the one below that is the best fit for your research. If you are not sure, read the appropriate sections before making your selection.

☒ Life sciences ☐ Behavioural & social sciences ☐ Ecological, evolutionary & environmental sciences

For a reference copy of the document with all sections, see nature.com/documents/nr-reporting-summary-flat.pdf

Life sciences study design

All studies must disclose on these points even when the disclosure is negative.

Sample size	No statistic tests were used to predetermine samples size. We follow the standards in the fields when choosing sample size for different experiments.
Data exclusions	Animals were excluded only if any health concerns existed. For the electrophysiological experiments, slices that demonstrated a series resistance was normally less than 20 MΩ) and recordings exceeding 20% change were excluded from analysis. For viral injection experiments animals were excluded if the injection was not in the correct region or there was no expression.
Replication	All experiments were replicated at least once, with concordant results.
Randomization	Mice were randomized to groups. Details are provided in the Methods.
Blinding	Experimentation and analyses were performed by experimenters in a manner completely blind to the treatment/experimental groups. Details are provided in the Methods.

Reporting for specific materials, systems and methods

We require information from authors about some types of materials, experimental systems and methods used in many studies. Here, indicate whether each material, system or method listed is relevant to your study. If you are not sure if a list item applies to your research, read the appropriate section before selecting a response.

Materials & experimental systems

n/a	Involved in the study	
☐	☒	Antibodies
☒	☐	Eukaryotic cell lines
☒	☐	Palaeontology and archaeology
☐	☒	Animals and other organisms
☒	☐	Clinical data
☒	☐	Dual use research of concern
☒	☐	Plants

Methods

n/a	Involved in the study	
☒	☐	ChIP-seq
☒	☐	Flow cytometry
☒	☐	MRI-based neuroimaging

Antibodies

Antibodies used

Parvalbumin (BM1339; BOSTER, Wuhan, China; 1:300) and secondary antibody (SA00013-1; Proteintech, Wuhan, China; 1:500)

Validation

All the commercial antibodies were validated as stated by the suppliers in their product datasheet, and can be easiliy accessed by using the catalog number and vendor information provided above.
The manufacturer's website:
mouse Anti-Parvalbumin
https://www.boster.com.cn/index/products/productsDetail?goods_sn=BM1339
CoraLite488-conjugated Goat Anti-Mouse IgG
https://www.ptgcn.com/products/CoraLite488-conjugated-Affinipure-Goat-Anti-Mouse-IgG-H-L.htm

Animals and other research organisms

Policy information about studies involving animals; ARRIVE guidelines recommended for reporting animal research, and Sex and Gender in Research

Laboratory animals

C57BL/6J and PV-Cre (Jackson laboratory#008069) mice on a C57BL/6 background 8-15 weeks old. All mice were housed under standard conditions at 22°C with 55% humidity and a 12h light: dark cycle with free access to food and water.

Wild animals

No wild animals were used in the study

Reporting on sex

Male and female pups were weaned and separately housed in groups of 3-5 with littermates who are the same treatment group until adulthood. Both male and female mice were used in our experiments, with nearly equal numbers in each group, as stated in the Methods section of our manuscript and in an additional table (Table S1 & S2).

Field-collected samples

There are no field collected samples

Ethics oversight

All experiments were conducted in line with the National Institutes of Health Guide for the Care and Use of Laboratory Animals, which were approved by the Animal Care and Use Committees of Ningbo University.

Note that full information on the approval of the study protocol must also be provided in the manuscript.

Plants

Seed stocks

N/A

Novel plant genotypes

N/A

Authentication

N/A
